# Examining the Relationship Between Resilience, Mental Health and Fitness Outcomes in Firefighters

**DOI:** 10.3390/jfmk10020142

**Published:** 2025-04-24

**Authors:** Daniel R. Greene, A. Maleah Holland-Winkler, Austin A. Kohler, William R. Kinnaird

**Affiliations:** Department of Kinesiology, Augusta University, Augusta, GA 30909, USA; awinkler@augusta.edu (A.M.H.-W.); akohler@augusta.edu (A.A.K.); wkinnaird@augusta.edu (W.R.K.)

**Keywords:** psychological, stress, anxiety, depression, PTSD, physical fitness, performance, tactical athlete, coping

## Abstract

**Background**: Firefighters have an increased risk of both mental and physical health conditions due to experiencing various forms of extreme stress regularly. High levels of resiliency may help firefighters overcome stressful situations and promote better mental and physical health. **Objectives**: The primary aim of the present study was to examine the relationship between resilience and other psychological variables. The secondary aim was to determine the relationship between psychological variables and firefighter fitness outcomes. **Methods**: Participants included 79 full-time male firefighters with a mean age of 35.9. They completed the following psychological questionnaires in this order: PTSD checklist for DSM-5, Dispositional Resilience Scale 15-item, State–Trait Anxiety Inventory for Adults and Beck Depression Inventory. They completed the following fitness tests in this order: maximum number of push-ups in two minutes, maximum time holding a plank and minimum time completing running and/or walking 1.5 miles. **Results**: Resilience was correlated with and predicted significant variance in depression, trait anxiety, state anxiety and PTSD symptoms in firefighters (all *p*’s < 0.025). Further, all psychological variables were significantly correlated with and predictive of each other. However, only scores on the Beck Depression Inventory were associated with push-ups completed (*p* = 0.014). No other psychological variable was related to fitness outcomes in firefighters. **Conclusions**: This study demonstrated resilience was significantly related to anxiety, depressive symptoms and PSTD symptoms in firefighters but not fitness outcomes. This highlights the protective effects of resilience on mental health, but future work needs to explore other psychological mechanisms to predict physiological performance variables in firefighters.

## 1. Introduction

Firefighters are at high risk for longer-term mental and physical health conditions due to the chronically and diversely stressful nature of their occupation [1]. Stressors include physical (e.g., intense workload, dehydration, dangerous working conditions and abnormal sleep) and psychological (i.e., rescuing those in need, safe and quick decision-making during emergencies and dealing with traumas that may or may not have occurred at the expense of their decisions while on call [2]). To endure and recover from these regular stressors, firefighters must be resilient. Resilience is the ability to withstand, recover and grow in the face of stressors and changing demands [3]. Resilience has been positively correlated with various aspects of health, including physical and mental health [4,5].

Resilience may potentially play a significant role in the ability of firefighters to continue in the occupation without developing psychological health conditions [6]. Previous research has demonstrated a negative relationship between the level of subjective resilience and the severity of PTSD symptoms in a sample of 116 professional firefighters [7]. It is also important to explore potential mediating factors between resilience and PTSD. For instance, anxiety and depression have significantly mediated the relationship between resilience and PTSD in 125 firefighter–paramedics [1].

A study by Zhang et al. demonstrated that higher levels of resilience were correlated to better emotional health in individuals compared to lower levels of resilience [8]. In addition, individuals that participated in higher levels of physical activity exhibited better emotional states and higher resilience compared to those that participated in lower levels of physical activity. Thus, those that are more resilient may participate in higher levels of exercise which may help reduce the risk for physical health conditions.

Firefighters are faced with significant physical challenges as part of their daily occupational demands. Among professional firefighters, job performance has been significantly correlated with total fitness, bench press strength, hand grip strength, bent-over row endurance, bench press endurance, shoulder press endurance, bicep endurance, squat endurance and 400 m sprint time [9]. Further, Michaelides et al. examined the predictability of specific fitness tests on firefighters’ performance via an ability test. Their results indicate that fitness tests significantly predict performance in firefighters [10]. As mentioned above, firefighters are exposed to significant physical tasks during shifts and it follows that higher levels of fitness correspond to an increased job performance; but, what predicts fitness levels in firefighters?

Previous research has identified psychological variables that may be effective at predicting physical fitness in firefighters. The Preference for and Tolerance of the Intensity of Exercise Questionnaire (PRETIE-Q; [11]) has been successful in predicting physical fitness in firefighters. Specifically, preference for exercise intensity was successful at predicting cardiovascular and muscular endurance while tolerance of exercise intensity nor extraversion were successful in predicting firefighter fitness [12]. This underscores the importance of examining psychological factors that can predict physical performance in high-risk groups like firefighters. Identifying and providing helpful health resources to improve psychological factors that influence firefighter performance and fitness may enhance the retention and overall effectiveness of firefighters within the profession.

Thus, the primary aim of the present study was to examine the relationship between resilience and other psychological variables in full-time firefighters. The secondary aim was to determine the relationship between psychological variables and firefighter fitness. Trait psychological measures including trait anxiety, depression, dispositional resilience and post-traumatic stress disorder (PTSD) symptomology were compared with physical fitness variables including push-ups, plank hold and a 1.5 mile run in firefighters. We hypothesized that: H1: Dispositional resilience would predict anxiety, depression and PTSD symptoms in firefighters. That is, higher resilience would be associated with significantly reduced anxiety, depression and PTSD symptomology. H2: Firefighters with greater dispositional resilience and lower trait anxiety, depression and PTSD symptoms would display greater muscular and cardiovascular endurance. H3: While anxiety, depression and PTSD symptomology are separate mental health conditions, it was predicted that each psychological condition would be significantly correlated with and predictive of each other in firefighters.

## 2. Materials and Methods

### 2.1. Experimental Design

A correlational, cross-sectional design was used to examine the relationship between objective physical fitness outcomes and subjective psychological scores in full-time firefighters in the Southeastern United States. All physical fitness and psychological measures were taken within the same month but on two separate days. This study was approved by the University’s Institutional Review Board (IRBNet ID: 1806526). All procedures followed institutional guidelines, and all participants signed informed consent.

### 2.2. Participants

Participants were recruited to volunteer via email and word of mouth. Participants included 79 full-time male firefighters who were at least 18 years old (ranged 19–58). All participants were employed by the same fire department which consisted of 185 employees including administrators. Participant demographics are included in Table 1.

### 2.3. Protocol

Physical fitness tests and psychological questionnaires were completed on two separate days within the same month in the fall of 2022. The physical fitness tests included a 1.5 mile run/walk test, plank hold and push-ups. Participants met at an outdoor 400 m track for the fitness tests in the morning. Before the physical fitness tests, they performed a structured dynamic warm-up, which included 2 × 6 squat sweeps, 2 × 6 reverse lunge rotation, 2 × 6 world greatest stretch, 5 yard Frankenstein and 5 yard hip rotation. After the warm-up, they completed the fitness tests in this order: push-ups, plank hold and 1.5 mile run/walk. Participants rested for 10 min between tests, and the rest was unstructured. The same firefighter trainer and study investigator led the fitness testing to reduce testing variability.

The psychological questionnaires were filled out in the morning at the participant’s fire station in a quiet room with the same investigator. To ensure confidentiality, the completed questionnaires were filed in a locked cabinet in a locked laboratory and only viewed by approved investigators. All fitness data and questionnaire data were recorded in a secure spreadsheet for subsequent analyses.

### 2.4. Physical Fitness Tests

#### 2.4.1. Push-Ups

Participants performed as many push-ups as possible in two minutes. Only push-ups performed with proper form were counted. The proper form included maintaining a straight line from head to heels throughout the motion with arms fully extended at the top of the push-up and chest just above the floor at the lower part of the push-up. The investigator recorded the number of push-ups for each participant.

#### 2.4.2. Plank Hold

Participants held a plank with proper form for the plank test for as long as possible. Proper plank form included maintaining a straight line from head to heels with hands directly under shoulders and arms extended. According to the investigator, once the participants dropped or the form was compromised, the timer was stopped, which signified the end of the test, and the investigator recorded the time.

#### 2.4.3. 1.5 Mile Run/Walk

Participants ran 1.5 miles (6 laps on a 400 m track) in the fastest possible time. Participants were allowed to walk during the test if needed. An investigator started and stopped the timer and recorded the time for each participant.

### 2.5. Psychological Questionnaires

The questionnaires were completed in the following order: PTSD checklist for DSM-5 (PCL-5), Dispositional Resilience Scale 15-item (DRS-15), State–Trait Anxiety Inventory for Adults (STAI) and then Beck Depression Inventory (BDI).

#### 2.5.1. PCL-5

The PCL-5 [13] is a 20-item self-report measure for assessing PTSD symptom severity. In some cases, it can be used to determine whether a person meets the criteria for PTSD. It is a less valid measure of diagnosis and more appropriate for assessing PTSD symptom severity. Participants respond to 20 different problems that represent stressful experiences by choosing the response that best fits how much they have been bothered by that problem in the past month, using a 5-point Likert scale ranging from 0 “Not at all” to 4 “Extremely”. The PCL-5 has demonstrated strong internal consistency (α = 0.94) and test–retest reliability (r = 0.82; [14]).

#### 2.5.2. DRS-15

Subjective resilience was assessed with the DRS-15. The DRS-15 is a 15-item measure of psychological hardiness related to commitment, control and challenge. It includes statements about life that people feel differently about, and participants were to rate how much they think each statement is true on a 4-point Likert scale ranging from 0, “Not true at all”, to 3, “Completely true”. The sum of the responses to the 15 items provides an overall resilience score with higher scores indicating greater hardiness and more likely to be resilient to stressors. The DRS-15 has shown good internal consistency (α = 0.82), 2 week test–retest reliability (α = 0.78) and criterion-related validity [15].

#### 2.5.3. STAI

The STAI is a 40-item inventory that measures both state and trait anxiety. 40 statements and participants indicated how they felt regarding those statements at the moment of completing the questionnaire on a 4-point Likert scale ranging from 1, “Not at all”, to 4, “Very much so”.

#### 2.5.4. BDI

The BDI is a 21-item inventory that assesses the severity of depression by evaluating related symptoms that they have felt during the past two weeks such as sadness, pessimism and worthlessness. Each item is scored on a 4-point Likert scale ranging from 0, “no impairment”, to 3, “severe impairment”, with higher scores indicating greater depressive severity [16].

### 2.6. Statistical Analysis

Statistical data analysis was conducted using SPSS (IBM Corp. Released 2021. IBM SPSS Statistics for Windows, Version 28.0.1.1, Armonk, NY, USA). Data were initially inspected for any unusual data points and missing data. Descriptive statistics were calculated to summarize demographic, psychological variables and fitness outcomes in firefighters. Pearson product–moment correlation coefficients were calculated to analyze the relationships between resilience and other psychological variables. Significant relationships (determined using an alpha level of 0.05) were further analyzed using hierarchical regression. Results are expressed as percentage change (i.e., R square change × 100).

## 3. Results

### 3.1. Descriptive Statistics

Firefighters in the present manuscript reported relatively low scores on the PCL-5, BDI and STAI. Participants can score from 0 to 80 on the PCL-5, with the average PCL-5 score being just 11.2 in the present sample. The BDI ranges from 0–63, with a mean BDI score of 9.2 in the present sample. Additionally, the TAI and SAI can range from 20–80, with firefighters in the present study reporting a mean of 38 and 37 for the TAI and SAI, respectively (see Table 2 for full descriptives on psychological variables and fitness outcomes).

### 3.2. Dispositional Resilience

Dispositional resilience was significantly correlated with state and trait anxiety, depression and PCL-5 scores (see Table 3). As such, hierarchical regression analysis was performed. Hierarchical regression revealed that the DRI accounted for 41.0 percent unique variance in TAI (*p* < 0.001), 25.4 percent unique variance in SAI (*p* < 0.001), 20.5 percent unique variance in depression (*p* < 0.001) and 7.0 percent unique variance in PCL-5 (*p* = 0.024).

### 3.3. Psychological Variables and Fitness Outcomes

To determine the relationship between psychological variables and physiological outcomes, bivariate correlations were conducted. Except for depressive symptoms being correlated with the number of push-ups completed (*p* = 0.014), no relationships between psychological variables (i.e., resilience, trait anxiety, state anxiety, depressive symptoms and PTSD symptoms) and physiological outcome (i.e., push-ups, plank hold and 1.5 mile run/walk) were identified (all *p*’s > 0.1). Depressive symptoms did account for 14.7 percent unique variance in push-ups completed (*p* = 0.014; see Table 3).

### 3.4. Predictability of Other Psychological Variables

Finally, we examined the relationship between all psychological variables. Bivariate correlations revealed significant relationships between all variables (trait anxiety, state anxiety, depressive symptoms and PTSD symptoms; see Table 3). Due to the strong relationships between each psychological variable, hierarchical regression analysis was performed. Hierarchical regression revealed that the TAI accounted for 67.6 percent unique variance in SAI (*p* < 0.001), 61.6 percent unique variance in depression (*p* < 0.001) and 35.8 percent unique variance in PCL-5 (*p* < 0.001). SAI accounted for 59.2 percent unique variance in BDI (*p* < 0.001) and 41.3 percent unique variance in PCL-5 (*p* < 0.001). Finally, BDI accounted for 34.9 percent unique variance in PCL-5 (*p* < 0.001).

## 4. Discussion

The purpose of the present manuscript was to examine the relationships between psychological variables and physiological outcomes in firefighters. Specifically, this study demonstrated the significant role resilience plays in the expression/display of trait and state anxiety, depressive symptoms and PSTD symptoms in firefighters. Further, this study showed that while resilience is highly related to psychological variables, it does not have an impact on performance variables. While the relationships between resilience and other psychological variables have been well documented in firefighters, there are few studies that assess these relationships with physiological outcomes.

First, it was hypothesized that dispositional resilience would be significantly correlated with and predictive of state anxiety, trait anxiety, depression and PTSD symptomology in firefighters. Correlation findings suggest that there is a significant relationship between resilience and other psychological conditions in firefighters. Specifically, firefighters who scored high in dispositional resilience reported significantly lower state anxiety, trait anxiety, depression symptoms and PTSD symptoms according to the STAI, BDI and PCL-5, respectively. Further, dispositional resilience accounted for 41, 25, 21 and 7 percent unique variance in TAI, SAI, BDI and PCL-5 scores, respectively. These results have been supported in the literature as resilience has been negatively correlated with anxiety, depression, job stress and PTSD symptoms in firefighters and firefighter–paramedics [1,7,17,18]. These results are also supported in other professions where individuals are exposed to high occupational stress. Police officers with lower resilience displayed significantly greater PTSD symptoms relative to police officers with higher resilience [19], and paramedics with low resilience experienced a significantly increase risk for PTSD, major depression and generalized anxiety disorder [20].

Resilience can be described in several related ways, including (1) the capacity to endure, recover and thrive when confronted with stressors and changing demands, (2) a psychological coping mechanism that enhances one’s ability to bounce back from adversity, (3) the ability to maintain positive adaptation in the face of hardship and (4) the ability to maintain a state of biopsychospiritual homeostasis following disruptive and adverse events [3,21,22,23]. Due to the occupational demands placed on firefighters, it follows that higher resilience would lead to a decrease in psychological conditions associated with traumatic events and high-pressure environments. The results of the present study confirm this and support existing research. Resilience was significantly correlated with anxiety and depressive symptoms in a sample of 225 firefighters [17] and served as a mediator in the relationship between exposure to traumatic events and depression in a sample of 7151 Korean firefighters [24]. Furthermore, while not significant, resilience was negatively correlated with depression and PTSD symptoms in firefighters [25]. This gives evidence that regardless of significance, resilience appears to be a consistent predictor of psychological distress in firefighters. Finally, to the best of our knowledge, no studies have identified resilience as a positive predictor for psychological distress in firefighters. Therefore, while higher resilience scores are typically associated with significantly lower symptoms of anxiety, depression and PTSD, no studies have found that higher resilience is linked to increased symptoms of these conditions.

Next, it was hypothesized that firefighters with greater dispositional resilience and lower trait anxiety, depression and PTSD symptoms would display greater muscular and cardiovascular endurance. Specifically, the firefighters that completed push-ups, plank holds, and a 1.5 mile run/walk for time. The results indicate that no significant relationships were present except for depressive symptoms predicting a significant percentage of push-ups completed. This is surprising and goes against the current hypothesis. While no clear relationships have been shown between psychological measures and physical fitness tests in firefighters, previous research has shown select psychological variables to be related to physical outcomes. Trait anxiety has been significantly correlated with agility, push-ups and step test repetitions in firefighters [26]. However, the same study failed to show a significant relationship between state anxiety and performance variables (i.e., agility, power, strength and endurance), and trait anxiety was not related to power, and numerous other strength/endurance tasks.

The finding that no relationship exists between the severity of psychological distress and physical performance in firefighters is noteworthy, but further exploration into this area is crucial for ensuring the safety and well-being of firefighters. Current literature is mixed on this topic with some studies showing select psychological variables to be directly related to physical fitness or performance and others showing no relationship. A recent study highlighted that firefighters’ exercise intensity preference predicted significant variance in cardiovascular endurance, muscular endurance and ability in recruit firefighters [12]. Further, numerous personality traits have been correlated with performance variables in firefighters. Specifically, conscientiousness, self-efficacy and openness were significantly correlated with strength and endurance, while extraversion was significantly correlated with functional movement in firefighters in training [27]. Interestingly, conscientiousness, openness and self-efficacy displayed a positive relationship with performance variables while extraversion was negatively correlated with functional movement. That is, individuals who scored higher in conscientiousness, openness and self-efficacy reported more positive performance outcomes, while individuals higher in extraversion experienced decreased functional movement [27]. Extraversion has also been negatively correlated with muscular endurance in recruit firefighters [12]. This is interesting as extraversion has typically been associated with an increase in exercise frequency and intensity [28]. Other studies have failed to identify any relationship between psychological variables and performance outcomes. A recent systematic review was unsuccessful at finding a single study looking at emotional or psychological variables associated with the Firefighter Physical Ability Test [29]. While this was a specific performance test, and other studies have examined psychological variables and physiological outcomes in firefighters, this review highlights a gap in the literature. Kim et al. showed that the amount of weekly physical activity may play a role in mental health in the general population [30]. Self-reports of mental health and hours of physical activity per week were collected and analyzed from 7674 adults during the 2008 U.S. Health Information National Trends 2007 Survey. The results revealed a dose–response relationship with 2.5 to 7.5 h of physical activity per week being the optimal volume for mental health benefits and poorer mental health with volumes less or greater than that range [30]. Thus, high volumes of physical activity may lead to improved physical fitness but not improved mental health. Repeating this study in firefighters may be beneficial to determine if the volume of weekly physical activity is playing a role on their mental health versus only assessing their physical fitness levels. Further, a recent review highlights the reciprocal relationship between resilience, PTSD symptomology, depression and anxiety in firefighters [31]. The relationships between resilience, psychological well-being and physiological outcomes in firefighters are clearly complex and warrant further investigation.

Finally, as firefighters are often exposed to traumatic events, occupational stress, and significant environmental risk factors, it was predicted that they would show a strong interrelation between anxiety, depression and PTSD symptomology. Specifically, it was hypothesized that each of these separate but related psychological conditions would be predictive of each other in firefighters. The results of the present study support this hypothesis. Trait anxiety, state anxiety, depressive symptoms and PTSD symptoms were all significantly correlated with each other. Further, trait anxiety scores predicted 68 percent, 62 percent and 36 percent unique variance in state anxiety, depressive symptoms and PTSD symptoms, respectively, in firefighters. State anxiety predicted 59 percent and 42 percent unique variance in depressive symptoms and PTSD symptoms, respectively, while depressive symptoms predicted 35 percent unique variance in PTSD symptoms. Overall, these results support the current literature and make sense logically as anxiety, depression and PTSD share common risk factors, exhibit symptom overlap, often occur comorbidly, may have a cyclical effect on one another and are exacerbated in high-stress environments (i.e., firefighting).

Prevalence rates for individuals living with PTSD, generalized anxiety disorder and major depressive disorder vary but are significantly higher in firefighters relative to the general population. The prevalence of PTSD symptoms in firefighters has been reported with rates ranging from 6.5% to 37% [32,33], relative to a lifetime prevalence of 5 percent in the general population [34]. Additionally, a recent report highlights the prevalence of anxiety and depression in firefighters to be significantly greater than the general population. More than 12 percent of firefighters met the criteria for anxiety, compared to only 6 percent of the general population, while just under 10 percent met the criteria for depression, in contrast to about 3 percent of the general population [35]. Furthermore, approximately 70 percent of firefighters with anxiety also met the criteria for depression, 27 percent with anxiety met the criteria for PTSD and 24 percent with depression met the criteria for PTSD [35]. In a sample of 200 firefighters, 57 percent met full DSM-IV criteria for PTSD symptoms; PTSD symptoms were significantly correlated with anxiety and depression, and between 44 and 54 percent of firefighters with PTSD also met the criteria for anxiety and depression [36]. Overall, firefighters are at an increased risk of PTSD, depression and anxiety symptoms. It appears that all three psychological conditions are linked, and individuals who experience symptoms of one are more likely to experience symptoms of the others. Thus, it follows that firefighters who experience a decrease in the symptoms of one psychological condition are likely to experience a decrease in other psychological conditions. It is important to further explore these topics and come up with specific interventions to decrease symptoms of PTSD, depression and anxiety in firefighters, as all are separate mental health conditions.

The present study is not without limitations. First, the sample of firefighters reported relatively low levels of anxiety, depression and PTSD symptomology. The average PCL-5 score was 11.18, and according to Weathers et al., scores of 33 or higher have been proposed as a potential threshold for a probable diagnosis [13]. The average trait anxiety and state anxiety scores were about 38 and 37, respectively. With a possible range of 20 to 80, scores between 20 and 37 are classified as “no or low anxiety”, scores from 38 to 44 are classified as “moderate anxiety” and scores from 45 to 80 are categorized as “high anxiety” [37]. The present sample was on the low end. Finally, the average score on the BDI was 9.22, which falls under the classification of “normal ups and downs”, which is the lowest (i.e., least symptomatic) classification of depression [38]. This is somewhat surprising as firefighters typically experience elevated anxiety, depression and PTSD, and limits the generalizability of the present results. Overall, the present sample has lower levels of anxiety, depression and PTSD symptomology, which may limit the generalizability of the study to other populations and professions. However, the relationships between psychological variables remained strong. It would be valuable to explore how psychological conditions predict physiological outcomes in a sample of firefighters with elevated or clinically significant levels of distress. Next, the present study did not explore mediation effects or cause-and-effect relationships. The primary aim was to examine the correlations among psychological variables and between psychological and physiological outcomes. A strength of the present study was its examination of psychological and physiological variables; however, future research is needed to deepen our understanding of these complex interactions and the mechanisms driving change.

## 5. Conclusions

Overall, the results of this correlational, cross-sectional study support previous research in that dispositional resilience continues to be a significant predictor of serious psychological conditions/symptoms (i.e., depression, PTSD, state and trait anxiety) in firefighters. That is, firefighters with greater dispositional resilience exhibit significantly lower symptoms of anxiety, depression and PTSD. Interestingly, these relationships were shown in firefighters with low levels of PTSD, depression and anxiety relative to both the general population and other first responders/firefighters. Additionally, the results revealed no significant relationships between psychological variables and fitness outcomes, except for a notable association between the BDI and the number of push-ups completed. While this goes against the original hypothesis, it might be worth exploring these relationships in a sample of firefighters with elevated anxiety, depression and PTSD symptomology. It may be that only elevated psychological distress is associated with fitness decrements. While most psychological variables were not significantly correlated to fitness outcomes, every assessment was in the hypothesized direction. That is, higher resilience and lower scores on anxiety, depression and PTSD symptomology were associated with more positive fitness outcomes (i.e., increased push-ups and plank hold, decreased 1.5 mile run time). Due to the cross-sectional design of the current study, establishing causality and generalizability is challenging, particularly given the low baseline psychological distress. Nevertheless, the findings support the use of resilience measures as a potential risk factor for firefighters. In other words, individuals with lower dispositional resilience may be more vulnerable to the negative psychological effects commonly linked to firefighting. Therefore, implementing interventions to boost dispositional resilience in firefighters with lower baseline scores could be beneficial. Future work is needed to fully understand the relationships between psychological variables and fitness outcomes in firefighters.

## Figures and Tables

**Table 1:** Firefighters’ demographics characteristics.

	Mean	SD
Age	35.53	9.2
Height (in)	70.7	3.04
Weight (lbs)	217.40	56.80
BMI	30.42	6.84

SD = Standard deviation; BMI = Body Mass Index.

**Table 2:** Descriptive statistics for psychological variables and fitness outcomes.

	Mean	SD
Psychological Variables		
Dispositional Resilience (DRS)	29.66	5.15
PTSD Symptoms (PCL-5)	11.18	14.22
Beck Depression Inventory (BDI)	9.22	7.30
Trait Anxiety (TAI)	37.92	9.84
State Anxiety (SAI)	36.59	9.87
Fitness Outcomes		
Push-ups (completed)	56.33	21.45
Plank hold (s)	146.88	73.14
1.5 mile run/walk (min)	16.49	4.64

SD = Standard deviation

**Table 3:** Correlation analyses between psychological variables and fitness outcomes.

Variables	DRS	TAI	SAI	BDI	PCL-5	Push-Ups	Plank	1.5 Mile
DRS	1.0	−0.640 **	−0.504 **	−0.453 **	−0.264 *	0.256	0.040	−0.193
TAI		1.0	0.825 **	0.785 **	0.598 **	−0.233	−0.140	0.192
SAI			1.0	0.770 **	0.642 **	−0.207	−0.179	0.247
BDI				1.0	0.591 **	−0.384 *	−0.125	0.173
PCL-5					1.0	−0.088	−0.229	0.178
Push-ups						1.0	0.639 **	−0.669 **
Plank							1.0	−0.667 **
1.5 mile								1.0

Note: DRS = dispositional resilience; TAI = trait anxiety; SAI = state anxiety; BDI = depression; PCL-5 = post-traumatic stress symptoms. ** Correlation is significant at the 0.01 level (two-tailed). * Correlation is significant at the 0.05 level (two-tailed).

## Data Availability

Data will be made available upon reasonable request from the corresponding author.
